# Prevalence of Micro-Aspiration of Bile Acids in Patients with Primary Lung Cancer: A Cross-Sectional Study

**DOI:** 10.4314/ejhs.v32i4.7

**Published:** 2022-07

**Authors:** Seyed-Mehdi Hashemi-Bajgani, Mitra Samareh-Fekri, Arshia Jamali Paghaleh, Rostam Yazdani, Mahboobe Asadi Zarandi, Ahmad Shafahi

**Affiliations:** 1 Afzalipour Hospital Research Center, Kerman University of Medical Sciences, Kerman, Iran; 2 Cardiovascular Research Center, Basic and Clinical Institute of Physiology, Kerman University of Medical Sciences, Kerman, Iran

**Keywords:** Aspiration, Bile acid, Lung cancer, Iran

## Abstract

**Background:**

Lung cancer remains a serious public health problem and is the first cause of cancer-related death worldwide. There is some evidence suggests that bile acid micro-aspiration may contribute to the development of lung diseases. This study aimed to assess the prevalence of micro-aspiration of bile acids in patients with primary lung cancer.

**Methods:**

In a cross-sectional study, 52 patients with primary lung cancer referred to a teaching hospital affiliated with Kerman University of Medical Sciences, Kerman, Iran were enrolled. Patients with pathology-confirmed lung cancer who did not receive specific treatment were included in the present study. All patients underwent bronchoscopy and the levels of bile acid was assessed in their Broncho-Alveolar Lavage (BAL) samples.

**Results:**

According to the results, 53.85% of patients were in the age group of 40 to 59 years. Of the participants, 88.46% were male, 82.69% were smokers, and 69.23% were opium addicted. The most common presenting clinical symptoms of patients were heartburn (61.55%), hoarseness (17.31%), and epigastric pain (9.61%), respectively. Ninety-two point thirty-two percent of patients had endobronchial lesions in bronchoscopy. Squamous cell carcinoma, small-cell lung carcinoma and adenocarcinoma accounts for 48.08%, 34.61% and 17.31% of all cases of lung cancer, respectively. Bile acids were found in the BAL sample of all patients with primary lung cancer. The mean Bile acids levels in patients were 63.42 (SD=7.03) µmol/Lit.

**Conclusion:**

According to the results of present study, there was a micro-aspiration of bile acids in all patients with primary lung cancer that may participate in shaping early events in the etiology of primary lung cancer. It seems that developing clinical strategies preventing the micro-aspiration of bile acids into the lungs could remove a key potential trigger in this process.

## Introduction

Lung cancer is one of the most common cancers worldwide. The disease is the cause of 29% of cancer-related deaths (1). Due to high relapse rate, late diagnosis and limitations in current treatment the prognosis in these patients is usually poor and 5-year survival rates are around 6.3% for men and 7.5% for women (2). Smoking and occupational exposure are the most important risk factors for this disease (3). Additionally, it has been shown that chronic obstructive pulmonary disease (COPD) is a major independent risk factor for lung cancer, so that between 50 and 70% of patients with lung cancer have COPD (4). Also, previous evidence has indicated that there is a link between gastro-duodenal esophageal reflux disease (GERD) and chronic pulmonary diseases (2–7). It has been shown that in patients with COPD the prevalence of GERD is high and exacerbates the negative consequences of this disease (8, 9). Also, non-acid reflux has been suggested as one factor for the chronic stimulation of lung tissue and reported its association with certain pulmonary diseases (10, 11). On the other hand, it has been shown that GERD has a higher prevalence in patients with lung cancer than in the general population (12, 13). The pathophysiologic relationship between reflux and pulmonary diseases has not been precisely defined. However, some cases such as vagus nerve stimulation in the esophagus and direct damage and chronic inflammation of airways and lung parenchyma from the micro-aspiration of gastro-duodenal secretions have been discussed (10, 11).

Bile acid aspiration has been recently suggested as a potential factor in developing complications in lung parenchyma and airways. It has been previously suggested that bile acids can regulate the immune response of airway epithelial cells by repressing HIF1 signaling through destabilization of HIF1-α and inducing the production of the pro-inflammatory cytokine interleukin 6 (IL6) (14, 15). The suppression of HIF-1 signaling by bile acids may have a significant influence on the progression and outcome of respiratory disease. Also, it has been shown that aspiration of bile acids could potentially cause cell damage, cell death and inflammation which can potentially contribute to the development of cancer (16). However, there is no convincing and conclusive evidence in this regard.

Therefore, due to the importance of this issue and considering that there is no dearth of evidence to evaluate bile acid micro-aspiration in patients with lung cancer, the present study aimed to assess the prevalence of micro-aspiration of bile acids in patients with primary lung cancer.

## Methods

**Study design and sample**: In a cross-sectional study, 52 patients with primary lung cancer referred to an educational-treatment hospital affiliated with Kerman University of Medical Sciences, Iran were enrolled. Data were collected from January to December 2014.

**Inclusion and exclusion criteria**: Patients with lung cancer who were confirmed by pathology and did not receive specific treatment were included in the present study. Dissatisfied participants were excluded from this study. Also, patients who could not have bronchoscopy were excluded from the present study.

**Data collection**: All patients underwent bronchoscopy using intravenous midazolam (2.5–5 mg) for sedation. Lidocaine with a concentration of 4% was used as the local anesthesia. To take Bronchoalveolar Lavage (BAL) sample (by a pulmonologist), 20 mL of normal saline was injected 3 times to the right middle lobe or right lower lobe of the non-involved lung, and the returned fluid was collected and frozen at -80°C. Samples were centrifuged at 1500 rpm for 10 minutes, and the obtained supernatant was evaluated for bile acid using the spectrophotometric enzymatic assay method using Total Bile Acid kit (BQ kits, San Diego, CA 92130). The bile acid assay was conducted according to the kit instructions. This kit has two solutions containing Thio-NAD and 3-α-hydroxy steroid dehydrogenase. The enzyme 3-α-HSD can convert bile acid to 3-keto steroids and Thio-NADH in the presence of Thio-NAD. Thio-NADH levels are measurable at 37°C using a spectrophotometer apparatus (405nm). Based on the related formula, bile acid levels were evaluated by a biochemist. Inasmuch as there is no accepted “normal” level of bile acid in BAL fluid, in this study micro-aspiration was defined as the presence of bile acids at any level in BAL samples that was detectable by spectrophotometric assay.

In addition, patients' demographic and clinical characteristics (such as age, sex, educational level, job, history of work experience with oven, smoking status, history of opium addiction, primary clinical symptoms and bronchoscopy findings and type of lung cancer) were evaluated and recorded.

**Sample size calculation**: The estimation of sample size was based on a presumed effect size of 0.3 (13), a statistical power of 95%, and a type I error of 5% using G*Power software, version 3.1.3 with the formula for calculation of sample of correlational studies. The overall proper sample size was found to be 52 participants.

**Statistical analysis**: Data were analyzed using the SPSS software package (version 16.0, SPSS Inc., Chicago, IL, USA). Mean (standard deviation) and number (percentage) were used to present quantitative and qualitative variables, respectively. The Kolmogorov-Smirnov test was used to assess the normality of the data. Chisquare and ANOVA tests were used to assess of relationship study variables. The significance level was set at *P*< 0.05.

**Ethical consideration**: The present study was confirmed by the ethics committee of Kerman University of Medical Sciences. The objectives of the research were explained to the participants and informed consent was obtained from them.

## Results

A total of 52 patients with primary lung cancer were enrolled in this study. 53.85% of patients were in the age group of 40 to 59 years. Of the participants, 88.46% were male, 40.38% were illiterate, 30.77% were farmers, 3.84% had work experience with the oven, 82.69% were smokers, and 69.23% were addicted (Table 1).

**Table 1:** Level of the micro-aspiration of bile acids based on individual characteristics and clinical features of patients with primary lung cancer (n=52)

	Total (n=52)	Level of the micro-aspiration of bile acids	*P*-value
***Individual characteristics***			
**Age (y)**			0.458*
**40–59**	28 (53.85)	53.81 (SD=11.49)	
**60–80**	21 (40.38)	53.87 (SD=12.44)	
**>80**	3 (5.77)	90.55 (SD=31.79)	
**Sex**			
**Male**	46 (88.46)	66.16 (SD=10.96)	0.442**
**Female**	6 (11.54)	42.41 (SD=4.41)	
**Level of Education**			
**Illiterate**	21 (40.38)	52.31 (SD=5.13)	0.789*
**Less than a diploma**	23 (44.23)	63.29 (SD=8.56)	
**Diploma**	8 (15.39)	35.56 (SD=3.32)	
**Job**			
**Farmer**	16 (30.77)	42.02 (SD=8.10)	
**Manual worker**	15 (28.85)	21.23 (SD=2.53)	0.879*
**Housewife**	5 (9.61)	34.52 (SD=5.33)	
**Employee**	6 (11.54)	29.26 (SD=5.61)	
**Freelance**	8 (15.39)	61.56 (SD=9.03)	
**Retired**	2 (3.84)	48.23 (SD=5.13)	
**Smoking**			
**Yes**	43 (82.69)	66.31 (SD=11.73)	0.523**
**No**	9 (17.31)	49.61 (SD=5.04)	
**Addicted**			
**Yes**	36 (69.23)	54.25 (SD=9.52)	0.247**
**No**	16 (30.77)	84.06 (SD=23.01)	
**Work experience with the oven**			
**Yes**	2 (3.84)	48.50 (SD=12.50)	0.763**
**No**	50 (96.16)	64.02 (SD=10.13)	
***Clinical features***			
**Clinical symptoms**			
**Hoarseness**	9 (17.31)	38.50 (SD=2.21)	
**Heartburn**	32 (61.55)	63.61 (SD=11.98)	
**Regurgitation**	4 (7.69)	44.00 (SD=9.30)	0.428*
**Dysphagia**	2 (3.84)	57.82 (SD=6.40)	
**Epigastric pain**	5 (9.61)	41.00 (SD=1.50)	
**Bronchoscopy findings**			
**Tracheomalacia**	1 (1.92)	31.32 (SD=4.31)	0.568*
**Endobronchial lesion**	48 (92.32)	61.92 (SD=8.65)	
**Abnormal secretion**	1 (1.92)	53.65 (SD=9.23)	
**Abnormal mucosal pattern**	1 (1.92)	42.32 (SD=4.36)	
**Bronchomalacia**	1 (1.92)	46.65 (SD=8.90)	
**Type of cancer**			
**Squamous cell c.**	25 (48.08)	74.12 (SD=16.76)	0.427*
**Small cell c.**	18 (34.61)	61.11 (SD=15.62)	
**Adenocarcinoma**	9 (17.31)	38.33 (SD=2.86)	
**Bile acid level (µmol/Lit)**	63.42 (SD=7.03)		

The most common clinical symptoms of patients were heartburn (61.55%), hoarseness (17.31%), and epigastric pain (9.61%), respectively. Also, 92.32% of patients had endobronchial lesions in bronchoscopy. Of the participants, 48.08% had squamous cell c., 34.61% had small cell c., and 17.31% had adenocarcinoma. Bile acids were found in the BAL fluid of all patients with primary lung cancer. The mean Bile acids in patients with primary lung cancer were 63.42 (SD=7.03) µmol/l. There was no statistically significant difference between the rate of micro-aspiration of bile acids and individual characteristics and clinical features in patients with primary lung cancer (*P*>0.05).

## Discussion

This study was assessed the prevalence of micro-aspiration of bile acids in patients with primary lung cancer. Regarding histological types, most of the tumor was squamous cell carcinoma (48.08%), followed by small-cell carcinoma (34.61%) and adenocarcinoma (17.31%). Bile acids were measurable in all BAL fluid of people with primary lung cancer. Bile acid is a component of duodenal secretions and logically should not be found in BAL fluid. Therefore, if bile acid is found in BAL fluid at any level, it is considered abnormal and indicates aspiration. However, the researchers had different criteria in this area (11, 17). A study in Canada showed that the normal level of bile acid in the BAL fluid of patients after lung transplantation is 0 to 8 µmol/L (11). Another study in Belgium showed that the presence of bile acids and pepsin in the BAL fluid of lung transplant patients is abnormal (17). There are diverse and complex mechanisms associated with the risk of developing lung cancer, COPD or indeed both. It has been previously indicated that COPD is a major independent risk factor for lung cancer (2, 3). Although the available evidence regarding bile acid micro aspiration and lung cancer is limited, there are some studies that evaluate bile acid micro-aspiration in patients with COPD. In a study by Hashemi-Bajgani et al. has been shown that in patients with COPD exacerbations the rate of bile acid micro-aspiration was higher than patients without COPD exacerbations, however this difference was not statistically significant (18). Another study revealed a significant higher concentration of total bile acids in tracheal aspirates in patients with ventilator-associated pneumonia (VAP) compared with patients without VAP (19).

Previous evidence has shown an association between GERD and many chronic lung diseases such as COPD (2, 5–7, 20–22). Not only the prevalence of GERD is higher in patients with COPD than in the normal population, but the severity of the disease, frequency and severity of relapses, length of hospitalization, and medication consumption rates are also higher in those patients (9, 23). The rate of GERD is also higher in patients with lung cancer than in the general population (12, 13). A study in the USA investigated the prevalence of GERD in 325 patients with lung cancer and 325 healthy individuals. The results indicated a significant higher prevalence of GERD in patients with lung cancer (23%), compared to control group (15%) (12). The pathophysiologic relationship between reflux and pulmonary diseases is still unknown. However, some pulmonary diseases can be a predisposing factor for reflux and, subsequently, aspiration. For example, different factors such as the use of inhaled or oral medications in COPD patients which causes lower esophageal sphincter (LES) relaxation and lungs hyperinflation, and flattening of the diaphragm that causes LES dysfunction and swallowing malfunction (24, 25). Furthermore, reflux and subsequently, micro-aspiration can create or exacerbate many pulmonary diseases. Reflux without aspiration can cause broncho-constriction and respiratory symptoms by stimulating the vague nerve in the esophageal plexus, but it seems the majority of adverse and dangerous effects of reflux on the respiratory system are caused by aspiration. The clinical effects of micro-aspiration on the respiratory system have been investigated mostly in patients with lung transplantation and idiopathic pulmonary fibrosis. These effects depend on factors such as the acidity of the aspirated liquid, the volume and frequency of aspiration, the existence of gastro-duodenal enzymes like pepsin and bile salts, and the ability to neutralize and clear the individual's respiratory system. Several studies have shown that the prevalence of gastro-duodenal micro-aspiration increases in lung transplant patients. It has also been found that pepsin as a protease in gastric secretions, compared with bile acid that is secreted from the duodenum, is a more sensitive criterion for proof of aspiration (11, 17, 26–28). A study in Canada showed that a higher bile acid concentration in BAL fluid in lung transplant patients is associated with the increasing early prevalence of bronchiolitis after transplantation. In addition, they showed that the increased level of bile acid in the BAL fluid of these patients is associated with an increasing level of inflammatory factors in the alveola, such as neutrophils, IL-8, and diminishing surfactant phospholipids (11). They also showed that bronchiolitis after transplantation is not only associated with the presence of bile acid in BAL fluid but related to its concentration. Based on these results, the researchers were convinced that gastroduodenal aspiration can cause pulmonary damage and the development of bronchiolitis after transplantation via increasing inflammation and decreasing intrinsic immunity. Therefore, it is considered a risk factor for transplant rejection (29, 30). Bile acid may induce alveolar epithelium permeability alteration, which may contribute to the pathogenesis of bile acid-associated lung injury (31).

In idiopathic pulmonary fibrosis (IPF), reflux and micro-aspiration are increased and they play a major role in the disease pathogenesis, progression, exacerbation, and survival of patients (32, 33). The results of a study by Aseeri et al. in patients with cystic fibrosis lung disease indicated that bile acids were measurable in all lungs of people with advanced disease, which was potentially damaging (34). Also, another study revealed an association between presences of bile acids in the lungs of patients with cystic fibrosis with alterations in the expression of inflammatory markers that is a predictor of the progression of structural lung disease (35).

It has been shown in animal models that chronic aspirations, regardless of the amount of acidity, can lead to parenchymal fibrosis (36). Bile acid can induce fibrosis of the airways with the production of TGF-β and increase the proliferation of fibroblast in epithelial cells (37). To identify the role of reflux and aspiration in the pathogenesis of IPF, the effects of reflux treatment on patients' symptoms and disease progression were assessed. Several studies have shown that the medical treatment of GERD can decrease the downward trend of FVC, improve survival, and decrease the exacerbation, although it does not affect the amount of coughing which is an important complaint of patients with IPF (38–43). A number of limitations in the present study deserve to be mentioned. The cross-sectional design of the current study limits our ability to form firm conclusions regarding causality. Also, lack of a gold standard diagnostic test for micro-aspiration of bile acid was another limitation of this study. Future studies are suggested to use liquid chromatography-tandem mass spectrometry, which can more accurately determine bile acids concentration.

In conclusion, according to the results of present study, there was a micro-aspiration of bile acids in all patients with primary lung cancer that may participate in shaping early events in the etiology of primary lung cancer. It seems that developing clinical strategies preventing the micro-aspiration of bile acids into the lungs or early clinical or surgical treatments for reflux could remove a key potential trigger in this process and alleviate bile-acid associated lung injury.
